# Cocultures of *Enterococcus faecium* and *Aeromonas veronii* Induce the Secretion of Bacteriocin-like Substances against *Aeromonas*

**DOI:** 10.1021/acs.jafc.3c04019

**Published:** 2023-10-02

**Authors:** Dusit Promrug, Kanjana Wittayacom, Nantipan Nathapanan, Ha Thanh Dong, Panumart Thongyoo, Sasimanas Unajak, Onrapak Reamtong, Usa Boonyuen, Amornrat Aroonnual, Tatsuo Shioda, Krit Thirapanmethee, Dumrongkiet Arthan

**Affiliations:** † Department of Tropical Nutrition and Food Science, Faculty of Tropical Medicine, Mahidol University, Bangkok 10400, Thailand; ‡ Faculty of Allied Health Science, Burapha University, 169 Long Had Bangsaen Rd, Saen Suk, ChonBuri District, ChonBuri 20131, Thailand; § Aquaculture and Aquatic Resources Program, Department of Food, Agriculture and Bioresources, School of Environment, Resources and Development, Asian Institute of Technology, Khlong Nueng 12120, Thailand; ∥ Department of Chemistry, Faculty of Science and Technology, Thammasat University, Phaholyothin Road, Klong Nung District, Klong Luang, Phatum Thani 12120, Thailand; ⊥ Department of Biochemistry, Faculty of Science, Kasetsat University, Chatuchak, Bangkok 10903, Thailand; # Department of Molecular Tropical Medicine and Genetics, Faculty of Tropical Medicine, Mahidol University, Bangkok 10400, Thailand; ¶ Department of Viral Infections, Research Institute for Microbial Diseases, Osaka University, 3-1 Yamadaoka, Suita, Osaka 565-087, Japan; ∇ Department of Microbiology, Faculty of Pharmacy, Mahidol University. 447 Sri-Ayuthaya, Rajathevi, Bangkok 10400, Thailand

**Keywords:** bacteriocins, lactic acid bacteria, Enterococcus faecium, Aeromonas, coculture

## Abstract

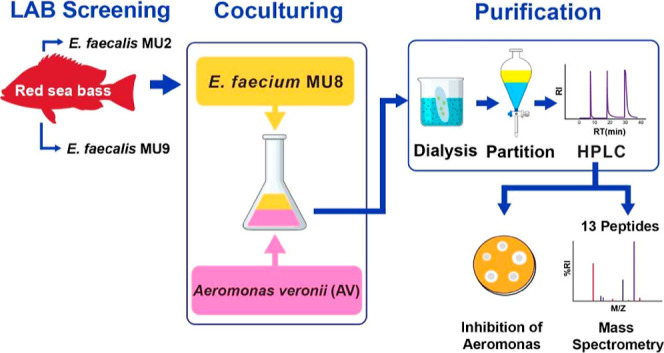

Lactic acid bacteria (LAB) were screened from *Lutjanus russellii* (red sea bass), and their antimicrobial activities were evaluated against two *Aeromonas* species isolated from the *Nile tilapia*, namely, *Aeromonas veronii* (AV) and *Aeromonas jandaei* (AJ). Three LAB isolates, *Enterococcus faecium* MU8 (EF_8), *Enterococcus faecalis* MU2 (EFL_2), and *E. faecalis* MU9 (EFL_9), were found to inhibit both AV and AJ; however, their cell-free supernatant (CFS) did not do so. Interestingly, bacteriocin-like substances (BLS) induced by cocultures of EF_8 with AV exhibited the highest antimicrobial activity against both *Aeromonas* sp. The size of BLS was less than 1.0 kDa; the purified BLS were susceptible to proteinase K digestion, indicating that they are peptides. BLS contained 13 identified peptides derived from *E. faecium,* as determined by liquid chromatography–tandem mass spectrometry. Cocultures of Gram-positive-producing and -inducing LAB strains have been used to increase bacteriocin yields. To our knowledge, this is the first report describing inducible BLS produced by cocultures of Gram-positive-producing and Gram-negative-inducing strains.

## 1 Introduction

*Nile tilapia* (*Oreochromis niloticus*) is a rapidly growing fish species that is commercially farmed in many countries, including Thailand. Tilapia is susceptible to infections caused by *Aeromonas* spp.^1,2^ and chronic osteomyelitis caused by *Aeromonas hydrophila* (AH), which results in hemorrhagic septicemia.^3^ In Thailand, *Aeromonas veronii* (AV) and *Aeromonas jandaei* (AJ) isolated from diseased *Nile tilapia* are pathogenic to tilapia juveniles, with a dose-dependent mortality rate.^4^

*Aeromonas* infections cause devastating economic losses for *Nile tilapia* in aquaculture worldwide. Therefore, preventive or curative measures are required for these diseases. To eliminate bacterial fish diseases in aquaculture, a wide range of conventional and advanced curative measures have been adopted. Lactic acid bacteria (LAB)-producing bacteriocins have been used as feed additives, dietary supplements, immunostimulants, prebiotics, and probiotics.^5–8^ LAB, which are commonly found in the gut microbiome of organisms including marine animals, play an important role in gastrointestinal (GI) tract development, digestive function, mucosal tolerance, stimulating the host immune response, and protection from infection.^8^ Bacteriocins are heterogeneous peptides or proteins with antimicrobial activities, which are produced by archaea and bacteria, including LAB.^9,10^ Bacteriocins that are produced by LAB usually exhibit cytotoxic activity primarily against other closely related bacteria.^10^

LAB-producing bacteriocins against a wide spectrum of potential fish pathogenic bacteria have been isolated from marine animals, such as European carps (*Cyprinus carpio*),^11^ spiny lobsters (Panulirus ornatus),^12^ gray mullets (*Mugil cephalus* L.),^13^ and Thai marine fishes.^14^ LAB isolates and their bacteriocins produced are suitable for widespread use as probiotics and biocontrol agents in aquaculture, respectively, as they are safe and effective.^13–16^

LAB-producing bacteriocins, which have been usually screened for putative antimicrobial substances or bacteriocins, are commonly used for disease control in aquaculture;^17,18^ however, these secretions have limited effects against the growth of pathogenic bacteria.^19^ Cocultures of a Gram-positive producer with a bacteriocin-inducing Gram-positive strain can increase the yield of bacteriocins and antimicrobial activity.^20–22^ For example, *Lactobacillus plantarum* KLDS1.0391 cocultured with any of the four different bacteriocin-inducing strains resulted in an increase in producer cell number and plantaricin MG production.^23^ In addition, cocultures of *Lactobacillus acidophilus* La-5 and *Streptococcus thermophilus* STY-31 increased lactacin B production.^22^ Plantaricin NC8 production was successfully increased by coculture in the presence of various Gram-positive bacteria.^24^ Thus, coculture techniques can improve the effectiveness of inducing or increasing the yields of bacteriocin for inhibiting the growth of target pathogens.

Because *Aeromonas* sp. causes significant economic damage to aquaculture, particularly in *Nile tilapia* farms, therapeutic treatments are desperately needed. Therefore, we screened LAB isolates producing bacteriocin-like substances (BLS) against *Aeromonas*, to find the advantageous cocultures in terms of BLS induction, including identification, characterization, and production optimization of the selected producer strains.

## 2 Materials and Methods

### 2.1 Bacterial Strains

The pathogenic bacteria, namely, AV and AJ, were isolated from *Nile tilapia* by Dong et al.^4^ AH was kindly provided by Dr. Unajak S. The three isolated LABs, *Enterococcus faecium* MU8 (EF_8), *Enterococcus faecalis* MU2 (EFL_2), and *E. faecalis* MU9 (EFL_9), were identified in this study. All bacteria were cultured in De Man, Rogosa, and Sharpe (MRS) (HiMedia, India) medium at a pH of 6.5 at 30 °C. The turbidity was measured by a spectrophotometer at 600 nm and calculated for the bacterial number.

### 2.2 Antimicrobial Assay

#### 2.2.1 Agar Spot Assay for Antimicrobial Activity

The screening of LAB exhibiting antimicrobial activity against *Aeromonas* (AV and AJ) using an agar spot assay^25^ was carried out. For bacterial screening, each LAB that was cultured and able to grow in MRS plates under anaerobic conditions was spotted on the new MRS agar and was grown for a further 3 days at 30 °C. Then, 70 μL of each pathogenic bacterium was mixed with 7 mL of warm, sterile MRS agar (semisolid form) and directly poured onto the MRS plate containing LAB. The MRS plates that were tested for fish pathogenic bacteria were incubated overnight at 30 °C. A vernier caliper was used for measuring the diameter (mm) of the LAB with an inhibition clear zone.

#### 2.2.2 Agar Well Diffusion Assay for Antimicrobial Activity

Cell-free supernatants (CFSs) or purified fractions were tested for bioactivity using an agar well diffusion assay.^26^ Each *Aeromonas* (AV or AJ) was diluted with normal saline solution (0.85%) to 0.5 McFarland standard (1.5 × 10^8^ cfu/mL). The adjusted cultured cells were then spread onto Mueller–Hinton agar plates. After the agar plates dried, wells were formed using a punch. Then 50 μL of the testing solution that was filtered through a 0.22 μm membrane filter was added to each agar well, and ampicillin was used as a positive control. The agar plates that were assayed for *Aeromonas* were incubated for 18 h at 30 °C. The diameter (mm) of the clear inhibition zone was measured using a vernier caliper. All experiments were done in triplicate.

### 2.3 Screening of LAB Isolates from Marine Fishes

Four dead *Lutjanus russellii* (red sea bass, size 10–20 cm) were obtained from a fishing port located in Chonburi province, Thailand, in December 2019. Fish intestines were collected and homogenized. The method for isolating LAB was modified from the procedure previously described by Chen et al. (2012).^27^ 1 g portion of homogenized fish intestines was mixed with 4 mL of normal saline (0.85% weight per volume) (w/v), and the mixture was serially diluted. Then, 1 mL of each diluted mixture was added to a methyl Petri dish plate. Warm, sterile MRS agar (semisolid) was poured onto the plate and mixed by gently swirling. All plates were incubated under anaerobic conditions (Anaerocult A system, Merck, Darmstadt, Germany) at 30 °C for 5 days. LAB colonies were selected from the MRS agar plates and further subcultured on the new MRS agar plates at 30 °C for 3 days. The subcultured LAB isolates were tested for antimicrobial activity against AV and AJ using the agar spot assay, as described in Section 2.2.1 All isolated LAB colonies were stored in a glycerol stock at −80 °C until use.

### 2.4 Identification and Characterization of LAB Isolates

#### 2.4.1 Strain Identification

The three LAB isolates were identified on the basis of their observed morphological characteristics by Gram staining as well as 16S rRNA gene sequencing.^28^ Bacterial genomic DNA was extracted by using a genomic DNA extraction kit (QIAGEN). Universal primers (Uni-Bact-F/AGA GTT TGA TCM TGG CTC AG and Uni-Bact-R/ACG GHT ACC TTG TTA CGA CTT) were used for the amplification of the 16S rRNA gene of the bacterial isolates. The polymerase chain reaction (PCR) mixture (25 μL) consisted of 0.5 nM each primer, 0.2 mM deoxynucleotide triphosphates (dNTPs), and 0.25 mM MgCl_2_), 1 U of Taq polymerase (Invitrogen), 100 ng of bacterial genomic DNA, and 19 μL of sterile water. The following thermocycling conditions were used: 94 °C for 5 min, 35 cycles of 94 °C for 40 s, 50 °C for 40 s, 72 °C for 1.5 min, and final extension at 72 °C for 7 min. Following gel electrophoresis, PCR products were stained with ethidium bromide, visualized under UV light, the target bands excised, and purified using the Favogen Gel/PCR Purification Kit following the manufacturer’s instructions. The 16S rDNA gene fragments were then cloned into the pGEM-T Easy vector (Promega), and the recombinant plasmids were sequenced. The sequence assembly was carried out using ContigExpress software and identified by the NCBI BLAST (http://blast.ncbi.nlm.nih.gov/Blast.cgi) search algorithm. The sequences were blasted against available nucleotide sequences in the GenBank database. A phylogenetic (neighbor-joining) tree was constructed using MEGA 6 software, and multiple alignments (Clustal W) of the 16S rRNA sequences from bacterial isolates and their closely related species were retrieved from GenBank.

#### 2.4.2 Growth Curve and Salt Tolerance

The LAB isolates were cultured in MRS medium overnight at 30 °C while being shaken at 220 rpm. 1 mL of each preculture was separately inoculated into 100 mL of MRS medium. The cultures were incubated at 30 °C and 220 rpm. Aliquots of the culture were taken at regular intervals, and the turbidity was measured by a spectrophotometer at 600 nm. The OD_600_ of the samples was recorded from 0 to 24 h. For the salt-tolerance test, LAB isolates were cultured overnight. Then, 200 μL of each preculture was inoculated in 2 mL of MRS medium containing salt concentrations ranging from 1, 3, 5, 8, and 10% (w/v), and then it was grown for a further 24 h at 30 °C and 220 rpm. The samples were collected at 24 h to measure their growth as described above.

### 2.5 Coculturing LAB Isolates with *Aeromonas*

The LAB isolates and *Aeromonas* were separately cultured overnight in MRS medium at 30 °C while being shaken at 220 rpm. Each culture was diluted to 0.5 McFarland in MRS medium. For coculturing each LAB isolate and each *Aeromonas*, 1 mL of an overnight culture of each LAB isolate and 1 mL of an overnight culture of each AV were mixed. CFSs were collected by centrifugation for 30 min at 10,000 *g* and 4 °C and then filtered through a 0.22 μm membrane filter. The antimicrobial activities of CFSs collected from the cocultured LAB with *Aeromonas* were tested using an agar well diffusion assay against AV. All experiments were done in triplicate. CFSs were collected from all LAB isolates, and *Aeromonas* exhibited no inhibition zone against AV (Figure S1). Among the three LAB isolates, coculturing the EF_8 LAB isolate with AV produced the most BLS against AV (Figure S2).

### 2.6 Optimization Conditions of the Bacterial Growth Phase and Ratios of Cocultures EF_8 and AV

The growth phase conditions for cocultures EF_8 and AV were optimized to induce BLS. Coculturing of EF_8 with AV was performed at a volume ratio of 1:1 (both cultures had equal cell numbers per volume). Initially, EF_8 and AV were cultured overnight in MRS medium at 30 °C and 220 rpm. Each culture was diluted to 0.5 McFarland in the MRS medium. For coculturing at the lag phase of growth, 1 mL of EF_8 and 1 mL of AV, each containing 0.5 McFarland of culture, were immediately mixed and grown for a further 24 h at 30 °C and 220 rpm. For coculturing at the log phase of growth, EF_8 and AV, each containing 0.5 McFarland of culture, were grown for 4 h at 30 °C while being shaken at 220 rpm. Then, they were mixed and grown for an additional 24 h at 30 °C and 220 rpm. For coculturing at stationary phases of growth, EF_8 and AV were grown overnight at 30 °C and 220 rpm. 1 mL of EF_8 and 1 mL of AV (equal cell numbers per volume) were mixed and grown for a further 24 h at 30 °C and 220 rpm. All CFSs were collected by centrifugation for 30 min at 10,000 *g* and 4 °C and then filtered through a 0.22 μm membrane filter. The antimicrobial activities of CFSs were tested using an agar well diffusion assay against AV. All experiments were performed in triplicate.

The ratios of coculturing LAB with AV for inducible BLS were performed at the log phase of growth. The volume ratios of EF_8 to AV (equal cell numbers per volume) were varied for 1:1, 1:2, 1:4, 1:8, 2:1, 4:1, and 8:1, respectively, and mixed in the final volume of 3 mL. Initially, EF_8 and AV were cultured in MRS medium overnight at 30 °C and 220 rpm. Then, they were diluted to obtain 0.5 McFarland and grown for a further 4 h at 30 °C and 220 rpm. After that, each 4 h cultured EF_8 and AV, for which volume ratios varied, were mixed. All coculture mixtures were grown for an additional 24 h at 30 °C and 220 rpm. All CFSs prepared from every condition were harvested by centrifugation at 10,000 *g* at 4 °C and filtered through a 0.22 μm membrane filter. All filtered CFSs were assayed for antimicrobial activity against AV using an agar well diffusion assay. All experiments were done in triplicate.

### 2.7 Isolation of Bacteriocins from CFS of LAB Isolate Cocultured with AV

#### 2.7.1 Large-Scale Preparation of Crude Bacteriocins

An overnight starting culture of EF_8 and AV was diluted to 0.5 McFarland in a total volume of 200 and 100 mL of MRS medium, respectively. Each culture was grown for a further 4 h at 30 °C with shaking at 220 rpm. Then, 200 mL of LAB isolate and 100 mL of AV were mixed and grown for a further 24 h at 30 °C and 220 rpm. CFS was harvested by centrifugation at 10,000 *g* for 30 min at 4°C. The CFS was filtered through a 0.22 μm membrane filter. After filtering, the CFS was freeze-dried and resuspended in 10 mL of sterile water before testing for antimicrobial activity against AV using an agar well diffusion assay.

#### 2.7.2 Dialysis

Dialysis of 20 mL of filtered CFS against 100 mL of sterile water was performed twice using a dialysis bag with a molecular weight (M.W.) cutoff of 1.0 kDa (Sartorius, Gottingen, Germany). The dialysate and dialysis water fractions were freeze-dried, resuspended in 10 mL of sterile water, and tested for antimicrobial activity against AV using an agar well diffusion assay.

#### 2.7.3 Methanol Precipitation

The active fraction obtained from the dialysis step (20 mL) was mixed with 60 mL of methanol (MeOH). The supernatant and precipitate fractions were collected by centrifugation at 10,000 rpm for 30 min. The precipitate was resuspended in 20 mL of sterile water. The supernatant was evaporated and lyophilized, and then it was resuspended in 20 mL of sterile water. Both the soluble fractions were then tested for antimicrobial activity against AV by an agar well diffusion assay. The soluble fractions of the supernatant were used for the further purification step for BLS isolation.

#### 2.7.4 Hexane/Acetonitrile Partition

The bacteriocin-containing supernatant was partitioned over five sequential rounds with hexane. The upper layer was pooled, dried, dissolved in dimethyl sulfoxide (DMSO), and assayed. The lower layer containing BLS was further partitioned with acetonitrile (ACN) for five rounds. The lower layer obtained from the ACN partitioning was pooled and evaporated. The upper layer was pooled, dried, and dissolved in DMSO. All fractions were tested for antimicrobial activity against AV by the agar well diffusion assay. The pooled upper phase was dissolved in 10% ACN prior to the high-pressure liquid chromatography (HPLC) separation.

#### 2.7.5 Preparative HPLC

The active fractions obtained from the partition step were purified by preparative HPLC using a C_18_ reverse-phase column with spectrophotometric detection at 215 and 280 nm (Jasco, USA). The samples were eluted using the following conditions: 5% ACN for 20 min, 18–32% ACN for 5 min, 32–42% ACN for 5 min, 42–60% ACN for 10 min, and 100% ACN for 15 min. The flow rate was 3 mL/min. Each fraction was collected based on retention time, evaporated, and lyophilized. All collected fractions obtained from preparative HPLC were tested for antimicrobial activity against AV by the agar well diffusion assay.

### 2.8 Characterization of Bacteriocins

#### 2.8.1 Mass Spectrometry Analysis

The peptides were enriched using reversed-phase C_18_ ZipTip chromatography (Millipore). The tips were prerinsed with 50% ACN. The peptides were resuspended in 0.1% trifluoroacetic acid (TFA) and loaded onto the tips. The samples were eluted with 0.1% TFA and 80% ACN. The peptides were dried in a speed-vac (Tomy, Tokyo, Japan). After resuspending in 0.1% formic acid, the peptide solution was injected into an UltiMateTM 3000 nano-LC system (Dionex, Surrey, UK). The column was an Acclaim PepMap RSLC 75 m, 15 cm nanoviper C_18_ (Thermo Scientific, Waltham, MA, USA). The LC system and MicroToF Q II mass spectrometer (Bruker; Bremen, Germany) were connected. A mass range between 500 and 3500 *m*/*z* was recorded. Data analysis was done using the MASCOT search engine 2.3 (Matrix Science, Chicago, IL, USA). A search was done using the Swiss-Prot database. The following search criteria were used: no enzyme, 0.8 Da peptide tolerance, 0.8 fragment mass tolerance, and 95% confidence.

#### 2.8.2 Effect of Heat on Bacteriocins

The BLS obtained from preparative HPLC, which was dissolved in sterile water, was heated at 100 °C for 30 min or autoclaved at 121°C for 15 min to assess the heat stability. The control included a BLS sample without heat treatment. All samples were tested for antimicrobial activity against AV by the agar well diffusion assay. All experiments were done in triplicate.

#### 2.8.3 Effect of Proteases on Bacteriocins

To evaluate the protease-tolerant property of bacteriocins, 50 μL of the BLS obtained from preparative HPLC, which was dissolved in sterile water, was treated with trypsin (0.1 mg/mL, Sigma-Aldrich, Germany) in 50 mM phosphate buffer pH 8.0 or proteinase K (0.2 mg/mL, Sigma-Aldrich, Germany) in 50 mM Tris-HCl buffer pH 7.5 for 5 h at 37 °C. A sample without protease treatment served as the control. The antimicrobial activity of the treated and control samples against AV was determined by using the agar well diffusion assay. All experiments were done in triplicate.

### 2.9 Statistical Analysis

The results were analyzed by a one-way analysis of variance (ANOVA) using the SPSS version 18.0 program. *p* < 0.05 was considered a statistically significant difference.

## 3 Results

### 3.1 Isolation of LAB from GI of *L. russellii*

The marine fish, *L. russellii* was screened for LAB isolates. 176 bacterial colonies were obtained from its gut. Three LAB isolates, C-2, C-8, and C-9, exhibited antimicrobial activity against AV and AJ (Figure S3).

### 3.2 Identification and Characterization of LAB Isolates

**Figure 1:**
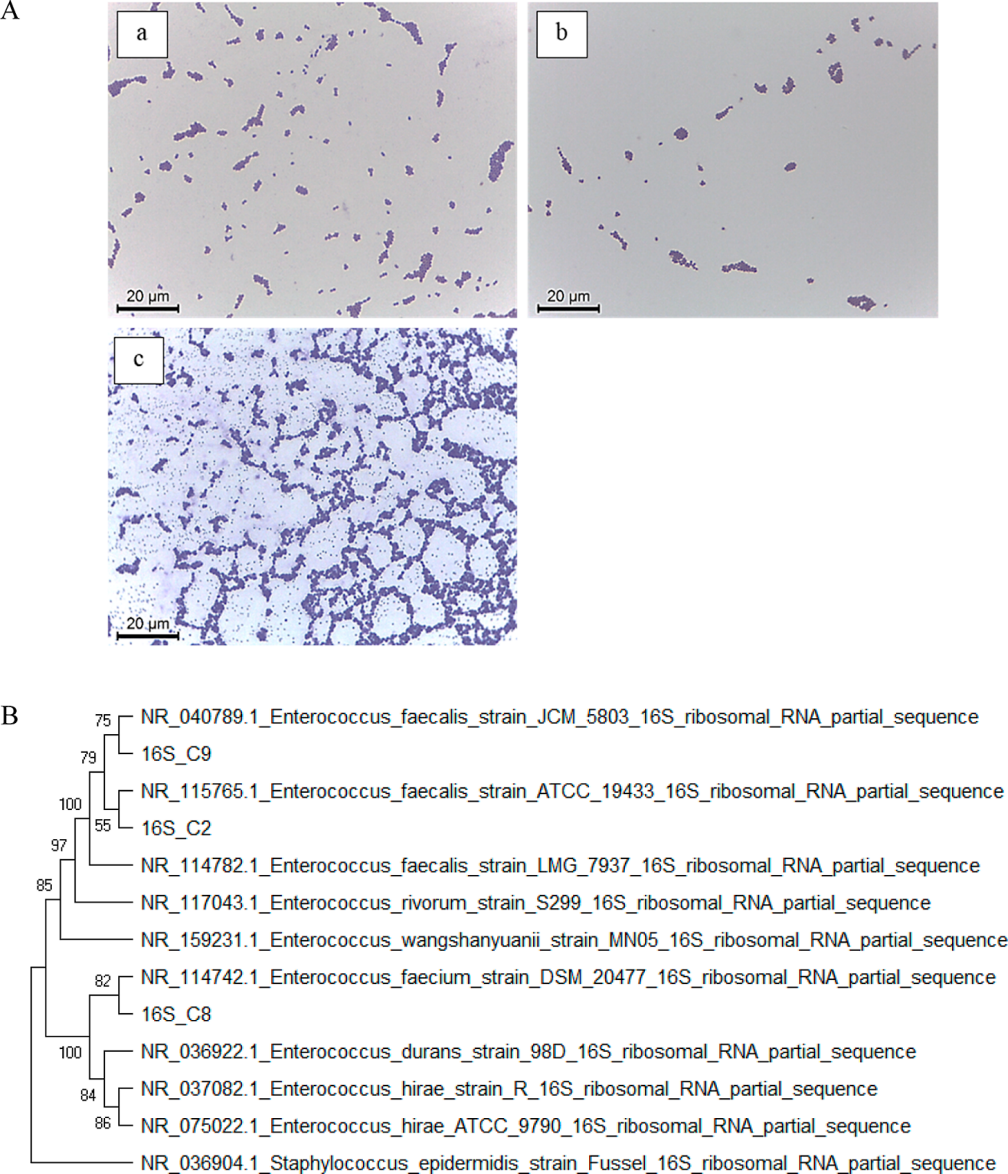
(A) Gram staining of LAB isolates (a) C-2; (b) C-8; and (c) C-9 (B) phylogenetic tree was constructed on the basis of 16S rDNA sequencing of LAB isolates from *L. russellii* and their closely related species. Percentage bootstrap values (1000 replicates) are shown at each branch point. The scale bar represents a 0.01 nucleotide change per nucleotide position.

**Figure 2:**
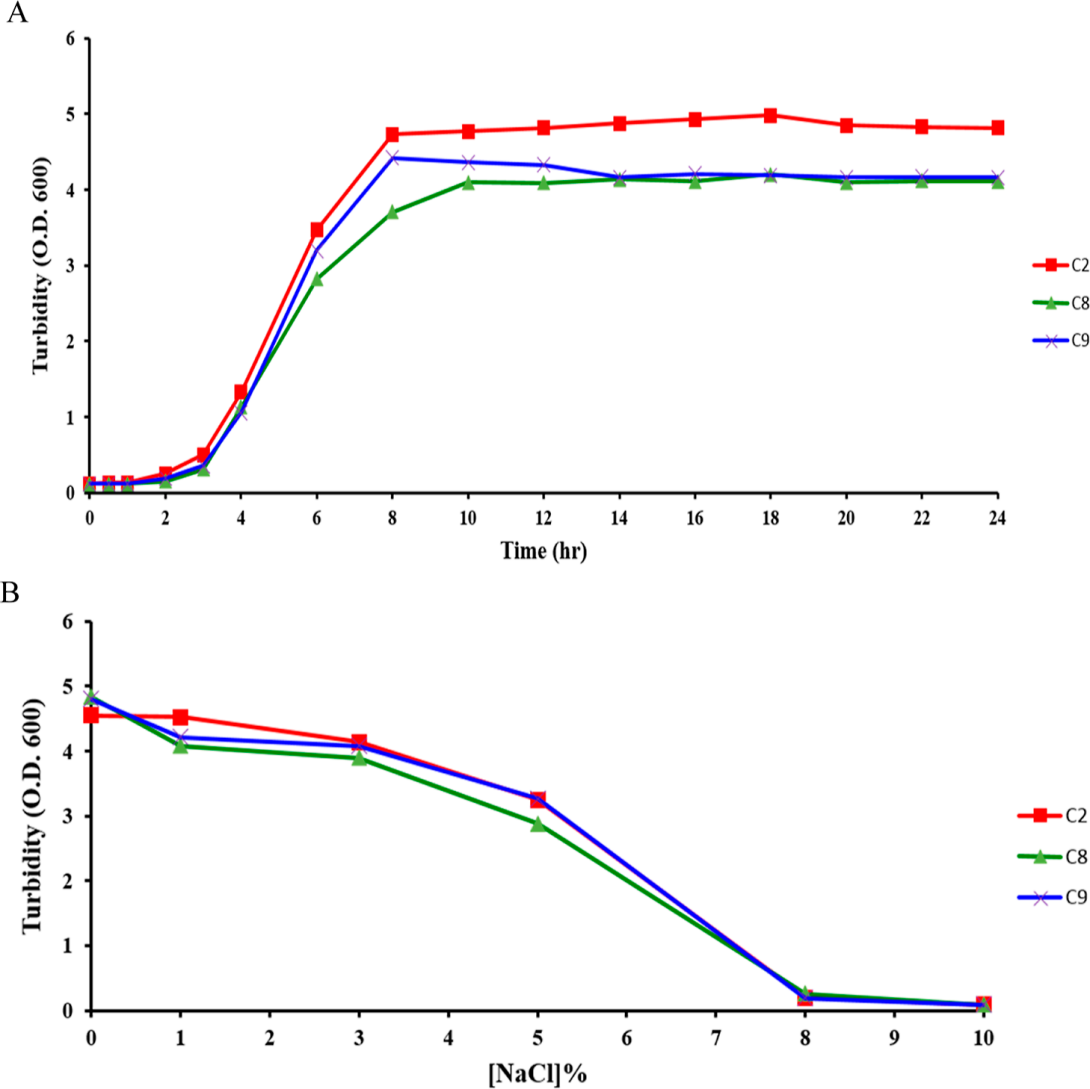
(A) Growth curve of LAB isolates and (B) salt-tolerance curve of LAB isolates.

Three LAB isolates, C-2, C-8, and C-9, were rod-shaped Gram-positive bacteria (Figure 1A). Amplification of 16S rRNA from these bacteria revealed an approximate 1.5 kb amplicon. BLAST results indicated that 16S rRNA amplified fragments of C-2, C-8, and C-9 isolates and the 16S rRNA sequence of C-8 exhibited 99.0% identity to *E. faecium* DSM 20477 (GenBank accession no: NR_114742), but showed a lower identity of 96.2 and 96.4% to the C-2 and C-9 isolates, respectively. The 16S rRNA sequences of both C-2 and C-9 exhibited 99.0% identity to *E. faecalis* LMG 7937 (GenBank accession no: NR_114782). Based on a combination of the homology of 16S rDNA sequences, a phylogenetic tree was constructed using the sequences of 16S rDNA of LAB isolates from the intestine of *L. russellii* and their closely related species. C-2 and C-9 isolates were identified and named as EFL_2 and EFL_9, respectively. C-8 was identified and named as EF_8 (Figure 1B). The growth curves of three LAB isolates (Figure 2A) revealed that EFL_2, EFL_9, and EF_8 exhibited a similar growth curve. All LAB isolates grew at the highest salt concentration of at least 8%; however, salt concentrations were increased. All LAB isolates grown at high salt concentrations showed similar curves, indicating that they all have the same level of salt tolerance (Figure 2B).

### 3.3 Optimization Cocultures of EF-8 and AV Conditions for Inducible BLS Synthesis

**Figure 3:**
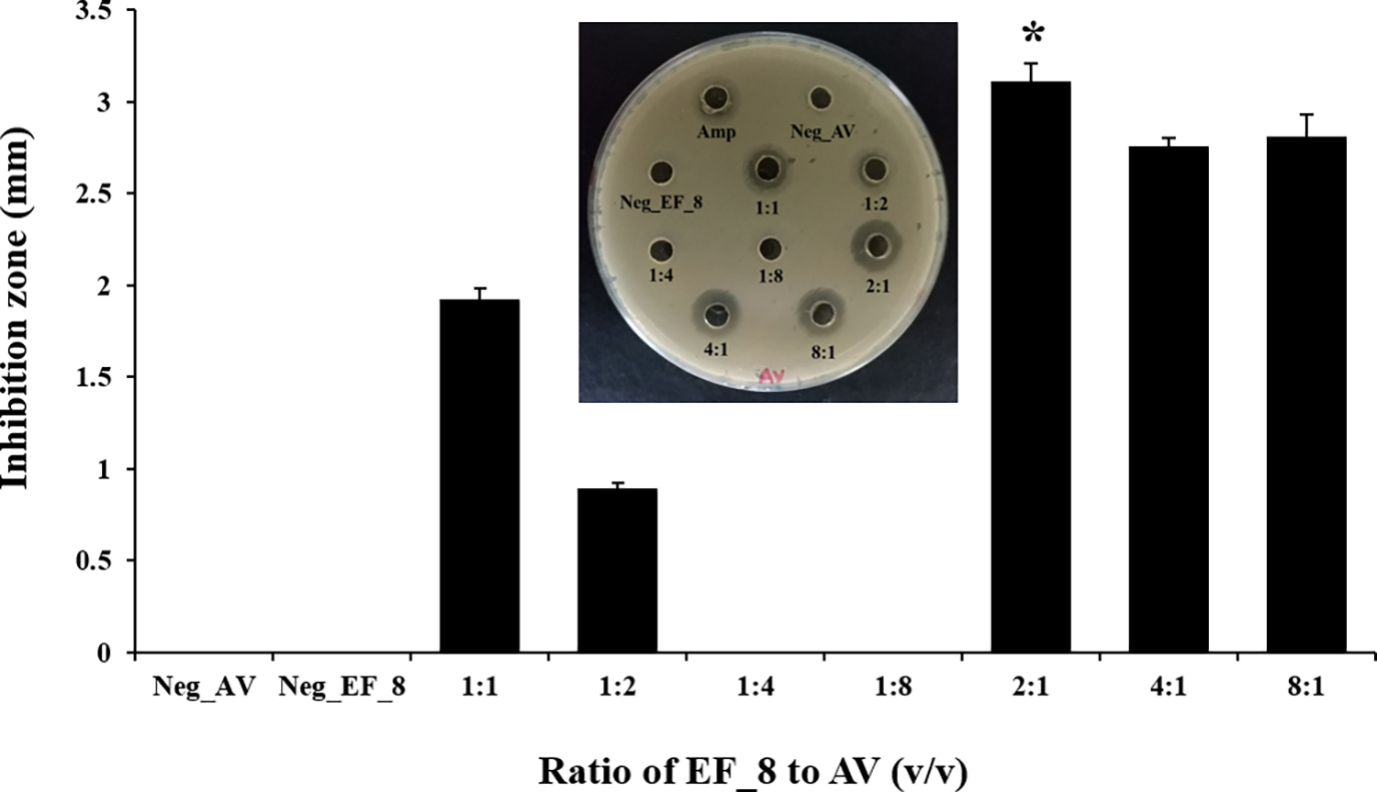
Optimization of volume ratio (equal cell number concentration) of EF_8 to AV for BLS secretion. All CFSs were assayed for antimicrobial activity against AV. Positive control: ampicillin. Negative controls; Neg_AV: CFS of AV; Neg_EF_8: CFS of EF_8. Note: The antimicrobial activity of BLS was determined using the agar well diffusion assay with AV as the indicator strain. Mean and standard deviation for *n* = 3. The experimental data were evaluated for significant differences by ANOVA. Significance at *P* < 0.05.

All CFS harvested from three LAB isolates, AV, and AJ exhibited no clear zones on AV and AJ (Figure S1). Among the three LAB isolates, the CFS of cocultured EF_8 with AV had the highest antimicrobial activities against AV (Figure S2). The growth phase conditions for cocultures of EF-8 and AV were optimized to obtain the highest yield of BLS. CFS of EF_8 cocultured with AV at the log phase showed inhibition zones against AV, but those at the lag and stationary phases did not (Figure S4). Furthermore, the volume ratio (v/v) of EF_8 cocultured with AV was optimized to obtain the highest bacteriocin at the log phase of the two bacteria species. The results indicated that a mixed volume ratio of EF_8 to AV (v/v) of 2:1 (equal CFU/mL) yielded the highest inhibition zone on AV (Figure 3).

### 3.4 Purification of BLS from CFS of Cocultures of EF_8 and AV

**Figure 4:**
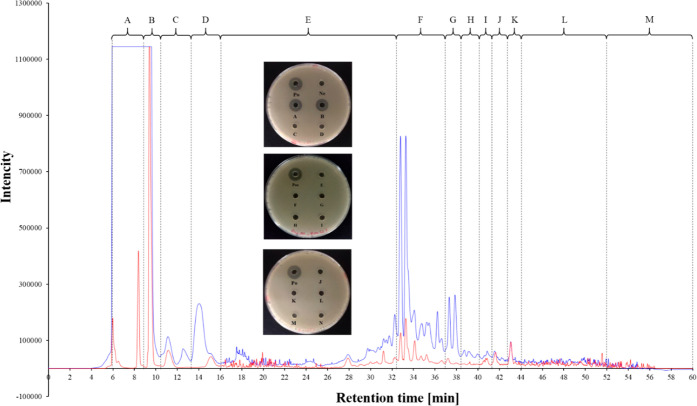
Chromatogram preparative HPLC of the active fraction from the hexane/acetonitrile partition step. Each fraction was collected according to the range of retention time. (A) Fraction of 5.7–9.1 min (B) fraction of 9.2–10.2 min (C) fraction of 10.3–13.2 min (D) fraction of 13.3–16.0 min (E) fraction of 16.1–32.3 min (F) fraction of 32.4–36.9 min (G) fraction of 37.0–38.2 min (H) fraction of 38.3–40.1 min (I) fraction of 40.2–41.2 min (J) fraction of 41.3–42.8 min (K) fraction of 42.9–44.0 min (L) fraction of 44.1–51.9 min (M) fraction of 52.0–60.0 min.

**Figure 5:**
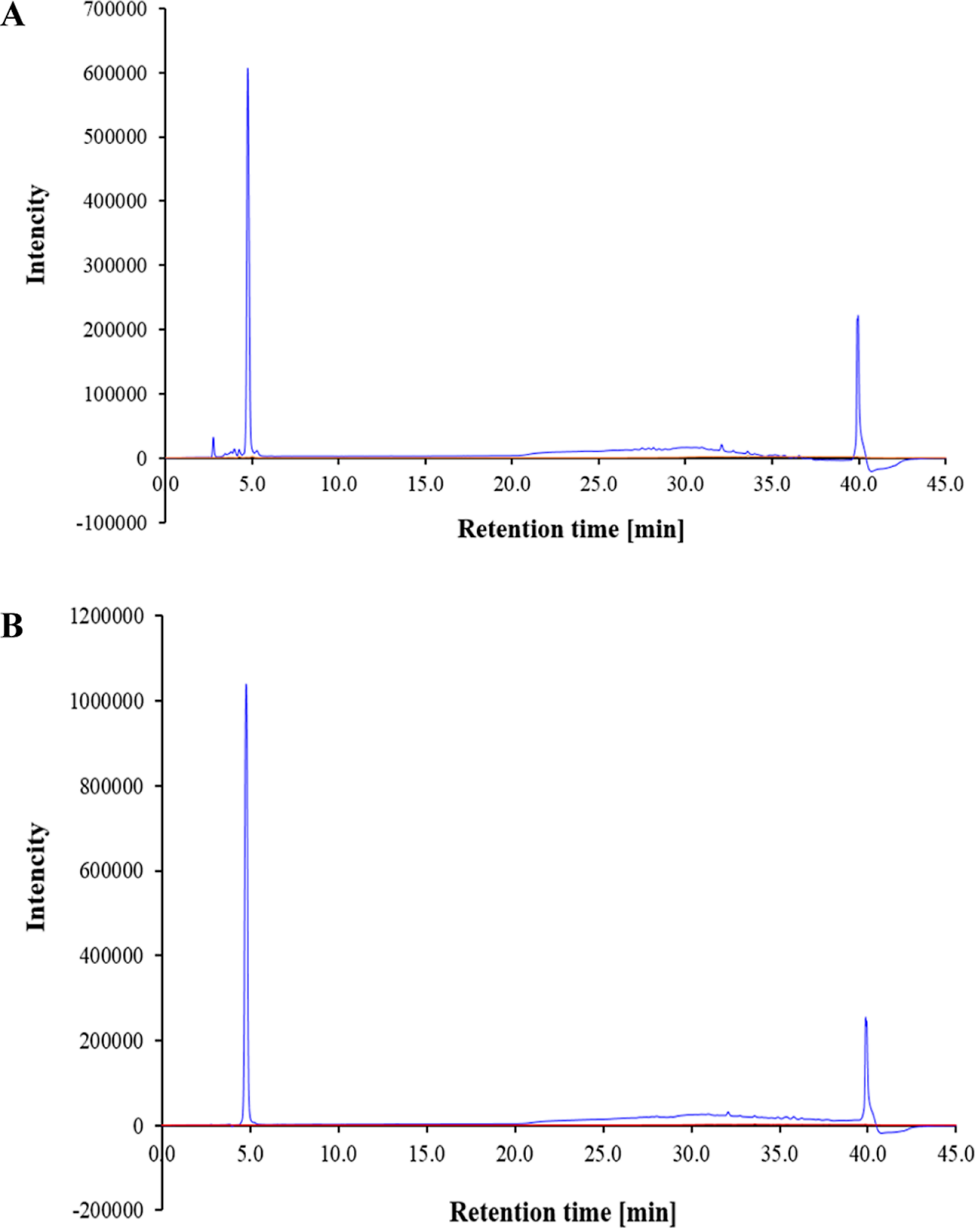
Analytical HPLC chromatography of the (A) pooled fraction of peak A and (B) pooled fraction of peak B.

CFS that was prepared from large-scale preparation, were dialyzed against sterile water using a dialysis bag with a M.W. cutoff of 1.0 kDa. The dialysis water fraction exhibited an inhibition zone on the AV, but not the dialysate fraction. Methanol was used to precipitate the active fraction obtained from the dialysis step. Only the supernatant fraction, but not the precipitate fraction, exhibited an inhibition zone on AV. The active fraction obtained from the methanol precipitation step was partitioned with hexane, yielding a lower layer with an inhibition zone on AV. The lower layer from the hexane step was further partitioned with ACN, and the lower layer exhibited an inhibition zone. The active fraction obtained from the hexane and acetonitrile partition steps was further purified by preparative HPLC. Each fraction was collected according to each peak, with a retention time range shown on the chromatogram (Figure 4). The fractions corresponding to peaks at the retention times of A-M were collected. The fractions from peaks A and B exhibited inhibition zones on AV and AJ, whereas no inhibition zone was observed in the other peaks (Figure 4). In addition, the active fractions obtained from peaks A and B were pooled because they showed the same retention time as those determined by HPLC analysis (Figure 5A,B). The purification of BLS was summarized, indicating that the final yield of the purification was 1.68% (Table 1). The inhibition zones on AJ and AV of the partially purified BLS were not significantly different but larger than those on AH (Table 2). The partially purified BLS fraction contained 13 identified peptides derived from *E. faecium* based on a liquid chromatography–tandem mass spectrometry (LC-MS/MS) analysis (Table 3).

**Table 1:** Summary of BLS Purification

steps	inhibition zones (mm/mL)	total volume (mL)	total inhibition zone (mm)	% yield	total weight^b^ (g)
CFS (after lyophilized)^a^	190	20	3800	100	15.9
dialysis	174	10	1740	45.8	10.9
MeOH precipitation	230	10	2300	60.5	3.59
hexane/acetonitrile partition	112	10	1120	29.5	2.98
preparative HPLC	64	1	64	1.68	0.04

a The 300 mL CFSs obtained from cocultures EF_8 and AV were used as starting materials for purification.

b All fractions obtained from each purification step were lyophilized and measured for total weight.

**Table 2:** Specificity of BLS for the *Aeromonas* Strains

*Aeromonas* strains	inhibition zone (mm)
AV	3.20 ± 0.07
AJ	3.17 ± 0.18
AH	2.17 ± 0.04^a^

a The antimicrobial activity of BLS was determined using the agar well diffusion assay with AV as the indicator strain. Mean and standard deviation for *n* = 3. The experimental data were evaluated for significant differences using ANOVA. Significance at *P* < 0.05.

**Table 3:** Peptide Candidates in the Active BLS Fraction Analyzed by LC-MS/MS

no	peptide names	peptide sequences	pI	charge	mass (Da)	hydrophobicity (%)
1	phage tail protein (*E. faecium*)	MTAILANLTK	8.50	1.00	1074.61	20
2	phage tail protein (*E. faecium*)	IPNNKHLTFY	8.60	1.09	1245.65	30
3	streptogramin A acetyltransferase (*E. faecium*)	IMNGANHRMDG	6.74	0.09	1246.51	36
4	oxidoreductase NAD-binding Rossmann fold protein (*E. faecium*)	MKILEEGIKGI	5.90	0.00	1245.70	36
5	hypothetical protein BH741_07110 (*E. faecium*)	HLMGKHIIAIN	8.76	1.18	1245.70	18
6	hypothetical protein CQR40_08150 (*E. faecium*)	IPNNKHITFY	8.60	1.09	1245.65	30
7	hypothetical protein EB09_02638 (*E. faecium*)	ISETIHKQKY	8.51	1.09	1245.67	50
8	hypothetical protein A5852_002501, partial (*E. faecium*)	LNNPAGSYAQPD	3.80	–1.00	1245.56	42
9	hypothetical protein A5804_002831 (*E. faecium*)	GKLMNHKKKY	10.18	4.09	1245.70	50
10	hypothetical protein A5852_002763 (*E. faecium*)	ADDQHYQV	4.20	–1.91	974.40	50
11	hypothetical protein A5852_002763 (*E. faecium*)	ADDQHYQVNSA	4.20	–1.91	1246.52	55
12	ATP-binding protein, partial (*E. faecium*)	AVMQHENM	5.24	–0.91	974.395	38
13	ATP-binding protein, partial (*E. faecium*)	AVMQHENMDR	5.32	–0.91	1245.52	50

### 3.5 Characterization of BLS

#### 3.5.1 Heat Stability of BLS

The inhibition zone on AV for samples heated at 100 °C for 30 min (2.83 ± 0.11 mm) or autoclaved (2.86 ± 0.07 mm) was not significantly different from that of unheated samples (3.06 ± 0.18 mm) (Table 4).

**Table 4:** Heat Stability of Purified BLS^a^

conditions	inhibition zone (mm) of AV
untreated sample	3.06 ± 0.18
heating 100 °C for 30 min	2.83 ± 0.11
autoclaved at 121 °C for 15 min	2.86 ± 0.07

a The antimicrobial activity of BLS was determined using the agar well diffusion assay with AV as the indicator strain. Mean and standard deviation for *n* = 3. The experimental data were evaluated for significant differences using ANOVA. *Significance at *P* < 0.05.

#### 3.5.2 Effect of Proteases on BLS

To determine the effect of protease treatment on BLS, inhibition zones on AV for proteinase K-treated BLS exhibited a significant difference compared to those of the untreated sample. However, the inhibition zones on AV for trypsin-treated BLS were not significantly different from those of the untreated BLS (Table 5).

**Table 5:** Effect of Proteases on BLS^a^

conditions	inhibition zone (mm) of AV
untreated sample	2.53 ± 0.06
trypsin	2.30 ± 0.15
proteinase K	0.00 ± 0.00*

a The antimicrobial activity of BLS was determined using the agar well diffusion assay with AV as the indicator strain. Mean and standard deviation for *n* = 3. The experimental data were evaluated for significant differences using ANOVA. *Significance at *P* < 0.05.

## 4 Discussion

LAB were screened from *L. russellii* (red sea bass), and their antimicrobial activities were evaluated against two *Aeromonas* species isolated from *Nile tilapia*, AV, and AJ. Three LAB isolates, EF_8, EFL_2, and EFL_9, inhibited both AV and AJ, but their CFS did not. Interestingly, BLS induced by coculturing EF_8 with AV exhibited the highest antimicrobial activity against both *Aeromonas* sp.

To optimize the conditions required to obtain the highest yield of BLS, cocultures of EF_8 and AV were mixed at the log phase of growth, and the volume ratio of EF_8 to AV was set at 2:1. CFS obtained from an EF_8 cocultured with AV was purified to identify BLS in four steps: dialysis, methanol precipitation, hexane/acetonitrile partition, and preparative HPLC using a C_18_ reverse-phase column. The fraction from preparative HPLC also exhibited antimicrobial activity against AV, AJ, and AH. The M.W. of BLS was less than 1.0 kDa because BLS can diffuse through a dialysis bag with a M.W. cutoff of 1.0 kDa. In the active BLS fraction, 13 identified peptide candidates derived from *E. faecium* were analyzed by mass spectroscopy. These 13 peptides have been potentially identified as the compounds to which the antimicrobial activity against *Aeromonas* may be attributed. These results also indicate that BLS was tolerant to heat because its microbial activity against the AV of heated BLS was only slightly decreased. The M.W. of BLS was less than 1.0 kDa, and the protein content of the active peptide substances was revealed by digestion with proteinase K, indicating that BLS contains peptides. The antimicrobial activity of BLS treated with trypsin against AV was unchanged, indicating that BLS isolated from coculturing EF_8 with AV is an active peptide containing a small number of arginine and lysine residues.

To date, there have been no reports of LAB isolates that inhibit the growth of AV and AJ. Several bacteriocins have been identified from *E. faecium*. *E. faecium* L3 cocultured with *Lactococcus lactis* yielded BLS with a M.W. > 5.0 kDa.^21^ Previously, *E. faecium* CTC492 isolated from fermented Spanish sausage was found to secrete enterocin A with a M.W. of 4.829 kDa,^29^ enterocin B with a M.W. of 5.465 kDa,^30^ and enterocin P with a M.W. of 4.493 kDa.^31^*E. faecium* C1 isolated from fermented cow milk secretes bacteriocin BacC1 with a M.W. of 10 kDa.^32^ BLS were compared with bacteriocins from another *E. faecium*. The size of BLS was less than 1.0 kDa and identified from cocultures of EF_8 and AV. These were obviously smaller than the known bacteriocins isolated from other *E. faecium* strains, indicating that the inducible BLS appeared to be novel.

Both the inhibitory spectrum and the biochemical properties did not provide sufficient information to identify the purified bacteriocin. Purification of bacteriocins to homogeneity is difficult and cumbersome because they are usually very small, polar, and post-translationally modified,^32^ resulting in high diversity as described in previous publications.^32,33^ Thus, based on our findings of peptide mixtures, it remains inconclusive whether BLS activities were caused by an individual or synergistic peptide. In addition, because of the limitations of mass spectrometry, it is unclear whether BLS are linear, modified, or cyclic peptides. Further studies are needed to synthesize each individual peptide and examine its microbial activity against *Aeromonas*.

*E. faecium* was reported as a probiotic that induces immune enhancers in olive flounder, which effectively controls lactococcosis.^16^ Diets supplemented with *E. faecium* improve the growth and health of *Arapaima gigas*.^17^ Because *E. faecium*_MU8 can inhibit fish photogenic bacteria, leading to probiotics application, *E. faecium*_MU8 mixed with fish feed may be a viable option for economic oral fish administration. They have high potential as probiotics administered orally to inhibit pathogenic fish bacteria, potentially replacing antibiotics. In fact, using pure bacteriocin for prebiotics is not required in the feeding industry. For prebiotic application, heated and filtered CFS from EF_8 cocultured with AV may be mixed with the fish feed of tilapia for *Aeromonas* prevention. Furthermore, high resistance to organic solvents, heat, and proteases are important industrial properties of BLS. Nonetheless, further studies in fish models are required to validate the potential application of BLS induced by the coculture of *E. faecium*_MU8 with AV.

Three LAB isolates were identified that exhibited antimicrobial activity against AV or AJ. Interestingly, CFS obtained from cocultured EF_8 with AV had the highest antimicrobial activity against AV and AJ. BLS with M.W. < 1.0 kDa were stable in organic solvents and heat-stable. These peptides exert anti-*Aeromonas* activity against both AV and AJ. *E. faecium*_MU8 are cocultured with AV and represent a useful option to control outbreaks of *Aeromonas* infections that cause devastating economic losses in aquaculture, particularly for *Nile tilapia*.

## Supporting Information Available

Inhibition zones against Aeromonas of CFS prepared from LAB isolates and Aeromonas, comparison of inhibition zones against AV of all CFS prepared from coculturing LAB isolates and AV, screening of LAB isolates against Aeromonas, and optimization growth phase conditions for coculturing EF_8 and AV (PDF)

## Abbreviations

AH: *Aeromonas hydrophila*
AJ: *Aeromonas jandaei*
ANOVA: analysis of variance
AV: *Aeromonas veronii*
BLS: bacteriocin-like substances
CFS: cell-free supernatant
LAB: lactic acid bacteria
M.W.: molecular weight
MRS: Man Rogosa and Sharpe
TRF: Thailand Research Fund
