# Supplementary material for: Cocultures of Enterococcus faecium and Aeromonas veronii Induce the Secretion of Bacteriocin-like Substances against Aeromonas
Source: J Agric Food Chem. 2023 Oct 2;71(43):16194–203. doi: 10.1021/acs.jafc.3c04019 (PMC10623555; doi:10.1021/acs.jafc.3c04019)
Supplement: Supplementary file 1 — jf3c04019_si_001.pdf [file jf3c04019_si_001.pdf]

Supporting Information

Cocultures of *Enterococcus faecium* and *Aeromonas veronii* induce the secretion of bacteriocin-like substances against *Aeromonas*

Dusit Promrug, Kanjana Wittayacom, Nantipan Nathapanan, Ha Thanh Dong, Panumart Thongyoo, Sasimanus Unajak, Onrapak Reamtong, Usa Boonyuen, Amornrat Aroonnual, Tatsuo Shioda, Krit Thirapanmethee, Dumrongkiet Arthan*

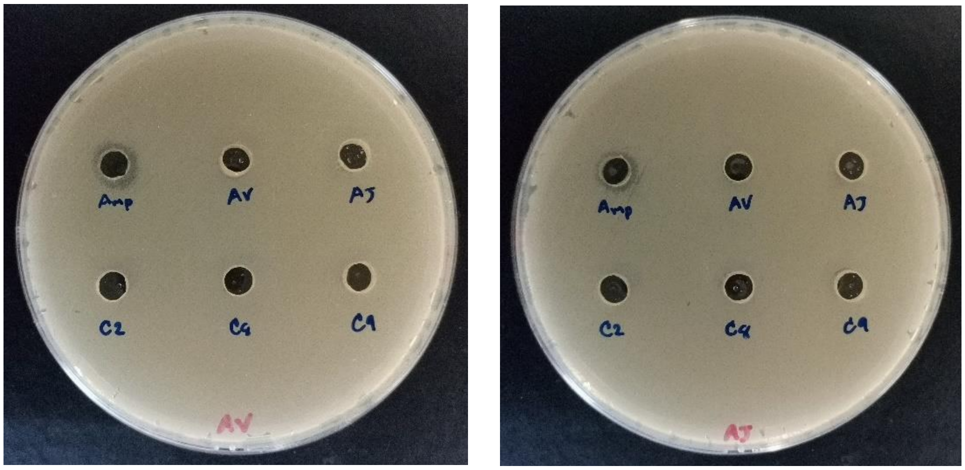

A B

Figure S1 Inhibition zones against *Aeromonas* of CFSs prepared from LAB isolates and *Aeromonas*. The antimicrobial activity was assayed against AV (A) and AJ (B). Positive control: ampicillin. Negative controls; AV: CFS of AV; AJ: CFS of AJ; C2: CFS of C2; C8: CFS of C8; C9: CFS of C9.

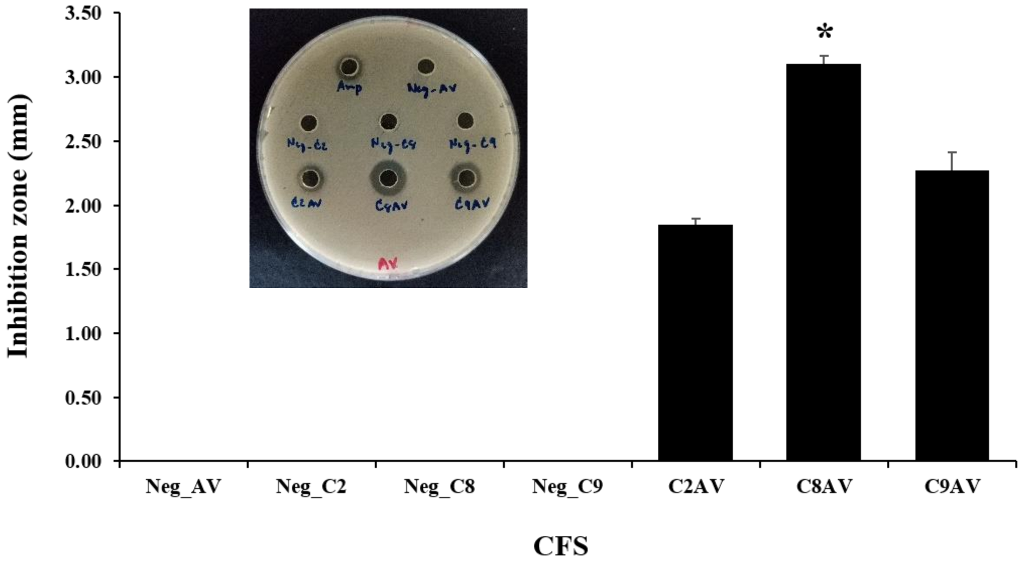

Figure S2 Comparison of inhibition zones against AV of all CFSs prepared from coculturing LAB isolates and AV. Positive control: ampicillin. Negative controls; Neg_AV: CFS of AV; Neg_C2: CFS of C2; Neg_C8: CFS of C8; Neg_C9: CFS of C9; C2AV: CFS of cocultures of C2 and AV; C8AV: CFS of cocultures of C8 and AV; C9AV: CFS of cocultures of C9 and AV.

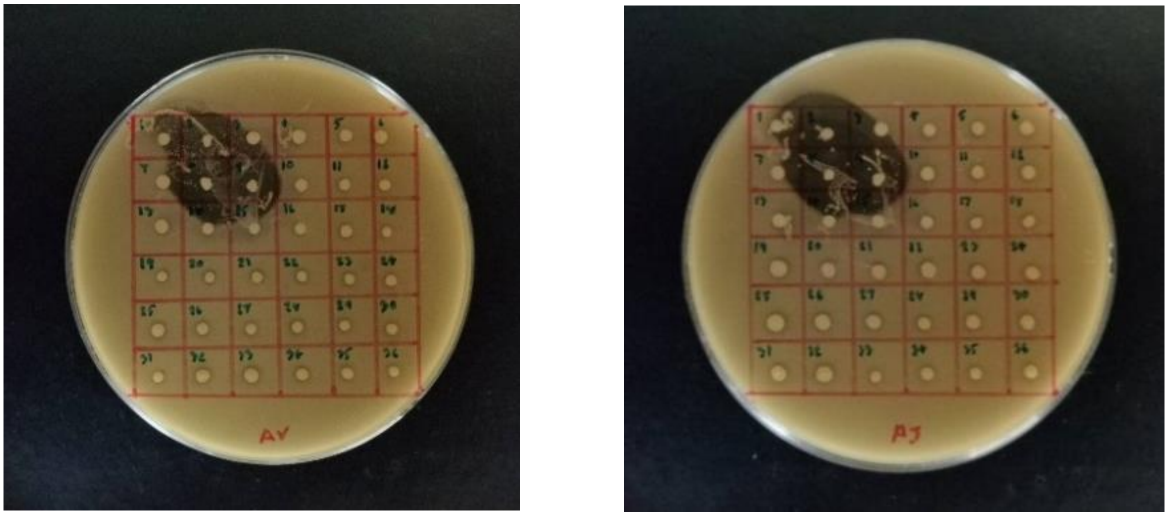

A B

Figure S3 Screening of LAB isolates against *Aeromonas* (A) *Aeromonas veronii* (AV) (B) *Aeromonas jandaei* (AJ).

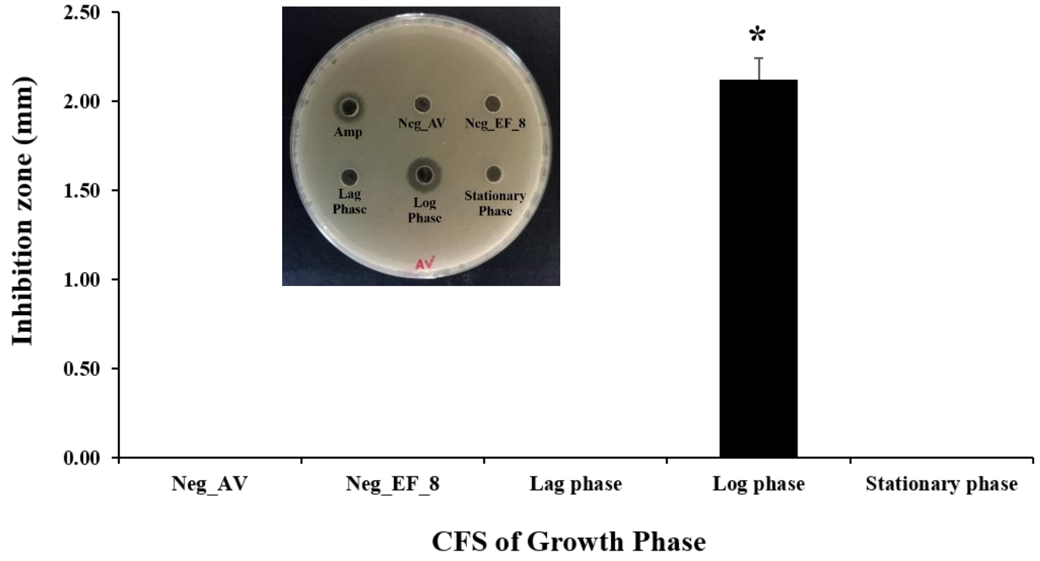

Figure S4 Optimization growth phase conditions for coculturing EF_8 and AV. Inhibition zones against AV. Positive control: ampicillin. Negative controls; Neg_AV: CFS of AV; Neg_EF_8: CFS of EF_8.
